# Temporal changes in key maternal and fetal factors affecting birth outcomes: A 32-year population-based study in an industrial city

**DOI:** 10.1186/1471-2393-8-39

**Published:** 2008-08-19

**Authors:** Svetlana V Glinianaia, Judith Rankin, Tanja Pless-Mulloli, Mark S Pearce, Martin Charlton, Louise Parker

**Affiliations:** 1 Institute of Health and Society, Newcastle University, William Leech Building, The Medical School, Framlington Place, Newcastle upon Tyne, NE2 4HH, UK; 2 School of Clinical Medical Sciences (Child Health), Newcastle University, Sir James Spence Institute, Royal Victoria Infirmary, Newcastle upon Tyne, NE1 4LP, UK; 3 National Centre for Geocomputation, John Hume Building, National University of Ireland Maynooth, Maynooth, County Kildare, Ireland; 4 Departments of Medicine and Paediatrics, Dalhouise University, Faculty of Computer Science, 6050 University Avenue, Halifax, Nova Scotia, B3H 1W5, Canada

## Abstract

**Background:**

The link between maternal factors and birth outcomes is well established. Substantial changes in society and medical care over time have influenced women's reproductive choices and health, subsequently affecting birth outcomes. The objective of this study was to describe temporal changes in key maternal and fetal factors affecting birth outcomes in Newcastle upon Tyne over three decades, 1961–1992.

**Methods:**

For these descriptive analyses we used data from a population-based birth record database constructed for the historical cohort **Pa**rticulate **M**atter and **P**erinatal **E**vents **R**esearch (PAMPER) study. The PAMPER database was created using details from paper-based hospital delivery and neonatal records for all births during 1961–1992 to mothers resident in Newcastle (out of a total of 109,086 singleton births, 97,809 hospital births with relevant information). In addition to hospital records, we used other sources for data collection on births not included in the delivery and neonatal records, for death and stillbirth registrations and for validation.

**Results:**

The average family size decreased mainly due to a decline in the proportion of families with 3 or more children. The distribution of mean maternal ages in all and in primiparous women was lowest in the mid 1970s, corresponding to a peak in the proportion of teenage mothers. The proportion of older mothers declined until the late 1970s (from 16.5% to 3.4%) followed by a steady increase. Mean birthweight in all and term babies gradually increased from the mid 1970s. The increase in the percentage of preterm birth paralleled a two-fold increase in the percentage of caesarean section among preterm births during the last two decades. The gap between the most affluent and the most deprived groups of the population widened over the three decades.

**Conclusion:**

Key maternal and fetal factors affecting birth outcomes, such as maternal age, parity, socioeconomic status, birthweight and gestational age, changed substantially during the 32-year period, from 1961 to 1992. The availability of accurate gestational age is extremely important for correct interpretation of trends in birthweight.

## Background

Maternal factors such as age and parity are known to influence birth outcomes. Thus advanced maternal age is associated with preterm birth [1-3], fetal loss and stillbirth [4-6], pregnancy complications [1], higher risk of perinatal mortality and low birthweight [7]. Higher risks of adverse outcomes are reported for both primiparous [1,8] and multiparous women of advanced maternal age (≥ 35 years) [1]. Birthweight and gestational age are, in turn, important predictors of perinatal and infant mortality [9,10], childhood morbidity and disability [11,12], and also health in later life [13,14]. The mutual interplay of the range of risk factors is complex and not yet fully understood.

While gestational age has been acknowledged as a major determinant of birthweight, it has not been collected as part of routine vital perinatal statistics in many countries, for example the UK [15]. Even when it has been included, it has been criticised for being inaccurate, in particular for singleton preterm births [16,17]. There is, therefore, a lack of information on long-term trends in gestational age alongside birthweight, making it impossible to meaningfully interpret temporal changes in birthweight. Other essential covariate information such as parity, mode of delivery and paternal and maternal occupation are also not routinely collected in the UK as part of national data.

The UK **Pa**rticulate **M**atter and **P**erinatal **E**vents **R**esearch (PAMPER) study offers the unique opportunity to describe temporal changes in key maternal and fetal factors affecting birth outcomes in a single conurbation over three decades, from 1961 to 1992. More specifically, we describe trends in maternal age, parity, aggregate level socioeconomic status, birthweight and gestational age and also demonstrate a reduction in stillbirth and infant mortality by decade.

## Methods

### Study setting

Newcastle upon Tyne, located within the Northern Region of England, has a current population of approximately 260,000 inhabitants. The population structure of the Northern Region is characterised by the low percentage of ethnic minorities, about 2% [18], and its relative stability with low levels of in and out migration. For example, among nearly 5,000 children aged between 1 and 11 years recruited into a study from 1996 to 1997, over 85% had lived at their address for most of their lives [19]. Residential mobility in pregnancy is also low: only 9% of cases notified to the population-based Northern Congenital Abnormality Survey (NorCAS) [20,21] moved from the time of booking to delivery (Rankin J, personal communication).

During the 50 years following the end of the Second World War, the economy of Newcastle transformed from one dominated by heavy industry and coal production and trade to a service based economy by the early 1990s. This paralleled remarkable changes in societal factors; for example, the 1967 Abortion Act, the National Health Service (Family Planning) Act (1967), availability of free family planning services irrespective of age or marital status from April 1974, the Sex Discrimination Act (1975) and the Employment Protection Act (1975) were introduced during the study period.

### PAMPER birth population

**Figure 1:**
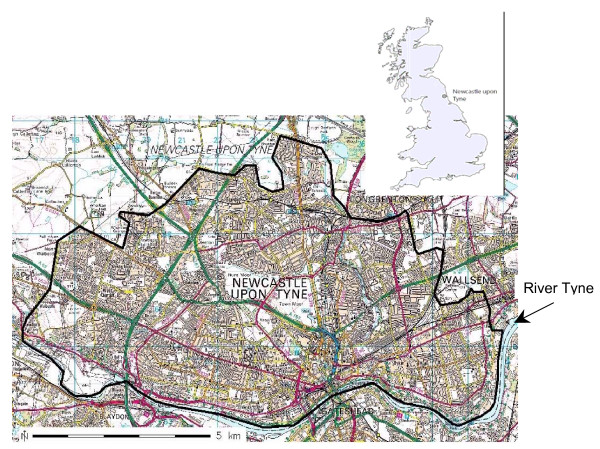
**Map of Newcastle upon Tyne with the PAMPER study area boundaries (black line)** (^© ^Crown Copyright/database right 2007. An Ordnance Survey/EDINA supplied service).

**Figure 2:**
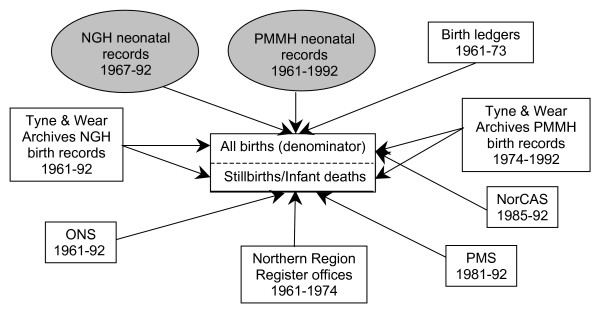
**Data sources used to construct the PAMPER dataset.** footnote: NGH = Newcastle General Hospital; PMMH = Princess Mary Maternity Hospital; ONS = Office for National Statistics; PMS = Northern Perinatal Mortality Survey; NorCAS = Northern Congenital Abnormality Survey.

The PAMPER database contains birth details on all singletons born during 1961–92 to mothers resident within the city of Newcastle upon Tyne in Northern England. Information on multiple births was also collected, however it was excluded from these analyses as multiplicity is a known risk factor for the outcomes of interest of the PAMPER study, i.e. preterm birth and low birthweight. The boundaries of the PAMPER study area are shown in Figure 1 with the river Tyne forming the southern boundary of the study area. The PAMPER computer database of birth records was constructed using information from a number of sources (Figure 2). The primary source was paper-based neonatal records from the two major maternity hospitals at the time (Princess Mary Maternity, PMMH, and Newcastle General Hospitals, NGH). From the PMMH, delivery and neonatal records were available for the whole study period, 1961–92; the NGH records were available from May 1967 onwards. These neonatal records contained information on important maternal and fetal/infant characteristics and clinical information about the delivery (Table 1). Socioeconomic information included paternal and maternal occupation, marital status and housing tenure.

**Table 1:** Key variables available across different data sources used for the construction of the PAMPER database

	**NGH and PMMH neonatal records**	**Tyne & Wear Archives birth records**	**Birth ledgers**
Mother's current surname	√	√	√
Residential address	√	√	√
Baby's sex	√	√	√
Date of birth	√	√	√
Vital status at birth	√	√	√
Place of birth	√	√	√
Plurality	√	√	√
Birthweight	√	√	-
Gestational age	√	√	-
Maternal age	√	√	-
Parity	√	√	-
Mode of delivery	√	√	-
Baby's surname	√	-	-
Paternal occupation	√	-	-
Maternal occupation	√ (for 1976–92)	-	-
Admission to Special Care Baby Unit	√	-	-
Resuscitation	√	-	-
Early mortality data with cause of death	√	-	-
Hospital morbidity data	√	-	-

**Note: **The following additional data on maternal and child characteristics are available in the PAMPER database either for the whole study period or for shorter periods: time of birth, date of discharge, discharge weight, date of death (in case of infant deaths), maternal blood group, marital status, housing (for the 1960s), details of previous births, placental weight, onset of labour (spontaneous vs induced), Apgar score, type of feeding on discharge, estimated date of delivery.

**Figure 3:**
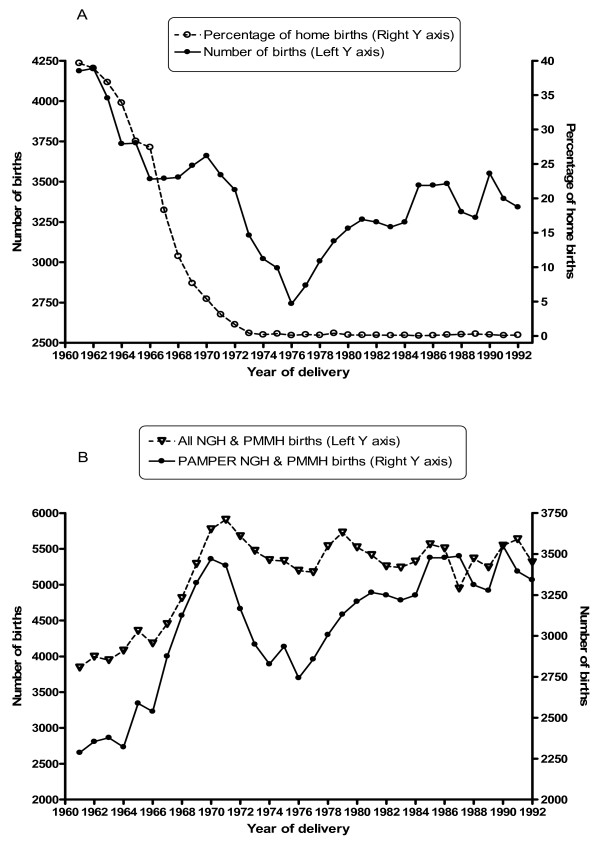
**(A) Number of births and percentage of home births by year of delivery (PAMPER dataset 1961–92) and (B) Number of hospital births by year of delivery in the PAMPER dataset and all births from two hospitals based on the Northern Region Health Authority data, 1961–92.** footnote: home births are recorded from both birth ledgers and hospital records for 1961–73 and from hospital records only thereafter; NGH = Newcastle General Hospital, PMMH = Princess Mary Maternity Hospital.

To capture home births, we additionally abstracted data from 'birth ledgers' (1961–1973), containing limited information on all births (Table 1). This data allowed us to obtain complete denominator information and to consider the changing proportion of home births (Figure 3A). We also used NGH birth records stored in the Tyne & Wear Archives (available from 24th April 1961), to complement information on key variables unavailable in birth ledgers (Table 1).

Each birth was georeferenced by postcode and/or grid reference. For births between 1961 and 1970 (prior to the introduction of postcodes), the address at birth was assigned a postcode from the 1991 postcode book or a grid reference. This allowed us to locate enumeration district (ED) of mothers' place of residence and hence to obtain the Townsend Deprivation Score (TDS), an area-based measure of material deprivation [22], at ED level (about 450 people in 200 households). TDS were calculated based on the 1971 (birth years 1961 to 1976), 1981 (1977 to 1986) and 1991 (1987 to 1992) Census data on unemployment, car ownership, owner occupation and overcrowding.

### Stillbirths and infant deaths

We linked the dataset to information on stillbirths and infant deaths (including causes of death) from the Office for National Statistics (ONS) and to death data from the Northern Perinatal Mortality Survey (PMS) (available from 1981 onwards) [23]. Multiple births were retained in the PAMPER database for the linkage procedure, but subsequently removed from the singletons database. Among a total of 1,248 eligible stillbirths provided by the ONS, we were able to match 1,222 cases (98%) to the PAMPER database. Among the total of 1,532 eligible ONS infant deaths, 1,510 (99%) were matched to the PAMPER database.

As Gosforth in the north and some western residential parts of the PAMPER study areas were not part of the city of Newcastle upon Tyne prior to 1974, the ONS could not provide us with all stillbirths and infant deaths for these areas for this earlier period. However, we obtained death certificates and causes of stillbirth for cases known to us to be stillbirths and infant deaths. This may still have resulted in some missing infant deaths if a postneonatal death was not recorded in the hospital notes.

### PAMPER database completeness and accuracy

Data entry staff (twelve individuals working 3-hour shifts) were trained in the medical terms/abbreviations used in the neonatal records and thus the percentage of errors was minimised. SVG was responsible for completing a descriptive 'summary' field, which contained the medical diagnosis and causes of death. In addition to the ONS and PMS data, stillbirth and infant death data were validated using birth record sources mentioned above.

At the initial stage of data entry, we double entered approximately 1% of the estimated total of 120,000 birth records for different decades of the study period (n = 1,474) to assess accuracy of the data entry results. At the final stage of data entry, the data were validated by checking for implausible values (e.g. implausible difference between date of discharge and date of birth, implausible birthweight by gestation combinations).

Table 2 shows that data derived from hospital records (97,809) had low percentage of missing values for the key variables. Table 2 also gives the number of births and percentages of maternal age, parity, birthweight, gestational age and mode of delivery categories by decade.

**Table 2:** Basic description of the PAMPER birth population 1961–92

**Variable**	**1961–70**	**1971–80**	**1981–92**	**N missing (%) **
				**1961–92**
**Maternal age (years)**				1796 (1.8)
Mean (± SD)	26.4 (6.2)	24.9 (5.2)	25.8 (5.3)	
≤ 19 [n (%)]	3037 (11.6)	4510 (15.1)	4913 (12.3)	
20–24 [n (%)]	8694 (33.2)	10676 (35.6)	12075 (30.3)	
25–29 [n (%)]	6772 (25.9)	9420 (31.4)	13123 (32.9)	
30–34 [n (%)]	4386 (16.8)	3917 (13.1)	7290 (18.3)	
35–40 [n (%)]	2426 (9.3)	1149 (3.8)	2175 (5.5)	
40–44 [n (%)]	807 (3.1)	259 (0.9)	287 (0.7)	
45+ [n (%)]	52 (0.2)	25 (0.1)	20 (0.1)	

**Parity **[n (%)]				1290 (1.3)
Parity = 0 (primipara)	10753 (41.1)	13659 (45.4)	17888 (44.4)	
Parity = 1	5673 (21.7)	9798 (32.6)	13209 (32.8)	
Parity = 2	3803 (14.5)	4121 (13.7)	5794 (14.4)	
Parity = 3	2101 (8.0)	1478 (4.9)	2114 (5.2)	
Parity = 4	1458 (5.6)	589 (2.0)	799 (2.0)	
Parity = 5	1004 (3.8)	225 (0.7)	278 (0.7)	
Parity = 6+	1383 (5.3)	201 (0.7)	191 (0.5)	

**Baby's birthweight (g)**				1360 (1.4)
Mean (± SD)	3244.2 (603.7)	3266.5 (540.3)	3297.0 (558.0)	
<1000 [n (%)]	150 (0.6)	75 (0.2)	128 (0.3)	
1000–1499 [n (%)]	270 (1.0)	181 (0.6)	238 (0.6)	
1500–1999 [n (%)]	481 (1.8)	354 (1.2)	496 (1.2)	
2000–2499 [n (%)]	1452 (5.6)	1445 (4.8)	1790 (4.4)	
2500–2999 [n (%)]	5132 (19.6)	6033 (20.1)	7504 (18.6)	
3000–3499 [n (%)]	9920 (37.9)	12175 (40.5)	15722 (39.1)	
3500–3999 [n (%)]	6719 (25.7)	7642 (25.4)	10852 (27.0)	
4000–4499 [n (%)]	1714 (6.6)	1907 (6.3)	3065 (7.6)	
4500+ [n (%)]	310 (1.2)	235 (0.8)	459 (1.1)	

**Gestational age (weeks)**				3562 (3.6)
Mean (± SD)	39.5 (2.2)	39.4 (1.9)	39.1 (2.0)	
< 32 [n (%)]	288 (1.1)	224 (0.8)	387 (1.0)	
32–36 [n (%)]	1533 (6.0)	1557 (5.3)	2415 (6.1)	
37+ [n (%)]	23769 (92.9)	27351 (93.9)	36723 (92.9)	

**Infant gender**				10 (0.01)
Male/Female ratio	1.08	1.06	1.07	

**Mode of delivery**				1763 (1.8)
Normal vertex delivery [n (%)]	20000 (76.8)	22517 (75.1)	29182 (72.9)	
Assisted (forceps/vacuum extraction) [n (%)]	3675 (14.1)	4502 (15.0)	5635 (14.1)	
Caesarean section [n (%)]	1822 (7.0)	2484 (8.3)	4670 (11.7)	
Breech extraction [n (%)]	543 (2.1)	470 (1.6)	506 (1.3)	

**Stillbirth **[n (rate per 1000)]	688 (18.2)	325 (10.5)	227 (5.6)	

**Infant mortality **[n (rate per 1000)]	770 (20.8)	453 (14.7)	281 (7.0)	

**Quintiles of ED TDS**				2095 (2.1)
1 (most affluent)	≤ 0.04	≤ -1.40	≤ -1.49	
2	> 0.04 to ≤ 3.01	>-1.40 to ≤ 2.43	>-1.49 to ≤ 2.18	
3	>3.01 to ≤ 4.9	>2.43 to ≤ 4.56	>2.18 to ≤ 5.06	
4	>4.9 to ≤ 6.2	>4.56 to ≤ 6.33	>5.06 to ≤ 7.04	
5 (most deprived)	>6.2	> 6.33	> 7.04	

**Address grid referenced**				1110 (1.1)

**Paternal occupation (coded)**				29419 (30.1)

**Note: **Number of births used for denominator = 109,086 (for calculation of stillbirth (per 1000 total births) and infant mortality rates (per 1000 live births); number of births from hospital records with information on covariates listed in the table = 97,809 (percentages of missing data are given using 97,809 as a total).

Percentages of the categories were calculated from the total with known data for a variable.

ED TDS = Townsend Deprivation Score at the enumeration district level.

For data capture we used the 4D database software suitable for a simultaneous data entry by several people, for data manipulation we used Microsoft Office Access 2003.

### Definitions

*Stillbirths *included were all babies born dead at 28 or more completed weeks of gestation. There were 12 cases (1%) recorded as stillbirths by the ONS with uncertain gestational age which were also included. Stillbirths with birthweight less than 500 g were excluded if gestational age was unknown. *Infant death *was defined as a death, following live birth, of an infant under one year of age. We defined *preterm birth *as birth at a gestational age less than 37 completed weeks and *term birth *as birth at a gestational age ≥ 37 weeks.

### Data analysis

For descriptive statistical analysis we used the statistical software package SPSS for Windows, version 14.0. We used chi-square tests to test differences in proportions and independent-sample t-tests for comparison of means.

### Ethical approval

The study received a favourable ethical opinion from the Sunderland Local Research Ethics Committee (SLREC 1071).

## Results

The number of births was highest in the early 1960s, followed by a steady decline until the mid 1970s and a further increase in the 1980s (Figure 3A). Home births constituted about a third of all births in the early 1960s, their proportion reduced to less than 0.5% by 1973 and remained low until the end of the study period (Figure 3A).

Figure 3B shows that the trends in the number of hospital births from the PAMPER data were in line with regional trends.

There was a dramatic decline in both stillbirth and infant mortality over the three decades (Table 2).

Between 1961 and 1992 the average family size decreased, mainly due to a decline in the proportion of families with ≥ 3 children (Table 2).

**Figure 4:**
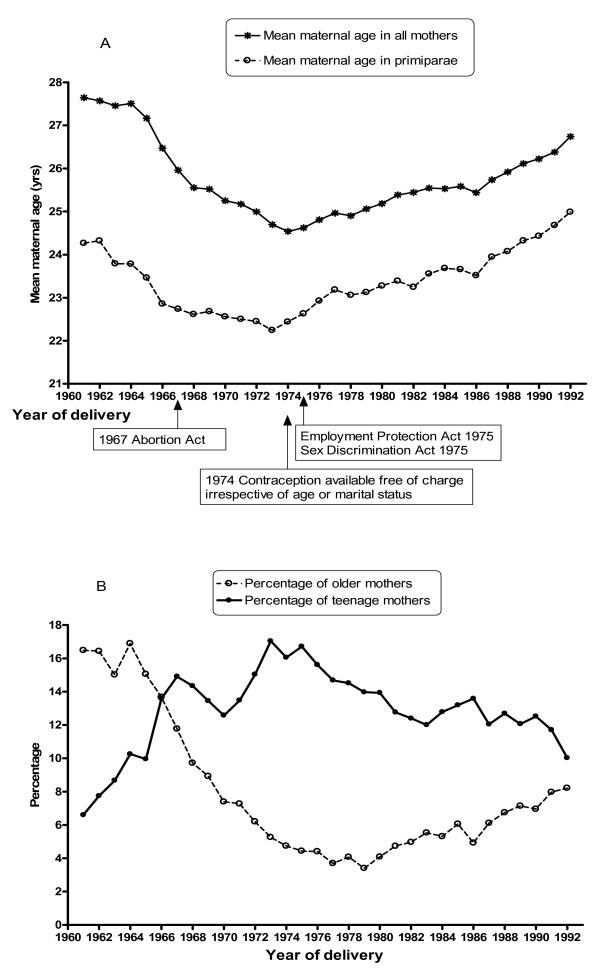
(A) Mean maternal age in all and primiparous women and (B) percentage of teenage (< 20 years) and older (≥ 35 years) mothers during 1961–1992.

We considered mean maternal age by year in all and primiparous women (Figure 4A) and the percentages of teenage (≤ 19 years) and older (≥ 35 years) mothers over time (Figure 4B) alongside a chronology of key legislative changes, which may have contributed to the observed temporal changes. The lowest mean maternal age corresponded to a peak in the proportion of teenage mothers in 1973. The proportion of older mothers declined until the late 1970s (from 16.5% to 3.4%) but this was followed by a steady increase.

**Figure 5:**
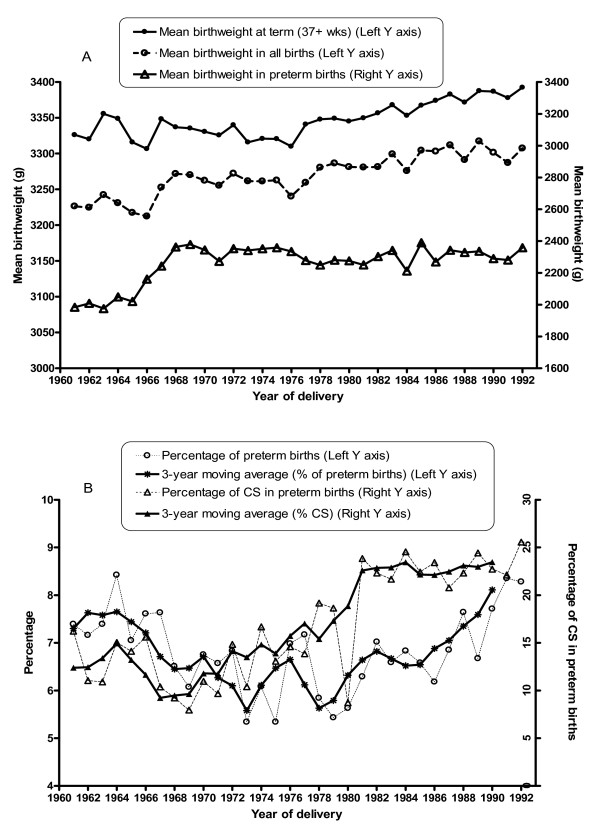
(A) Mean birthweight in term (≥ 37 weeks), preterm (<37 weeks) and all births by year of birth; (B) Percentages of preterm birth and caesarean section (CS) among preterm births by year of birth and respective 3-year moving averages of the percentage.

Mean birthweight was lowest in the early 1960s, averaging around 3267 g in the second decade, followed by a gradual increase during the second half of the study period (Figure 5A and Table 2). The increase in mean birthweight for term births mostly accounted for the overall increase in mean birthweight, in particular in the last decade (Figure 5A). Thus during 1981–92 the mean birthweight at term [3373 g (SD ± 472)] was significantly higher than during the first two decades [3333 g (SD ± 497) in 1961–70 and 3332 g (SD ± 465) in 1971–80, *p *< 0.001], whereas the mean birthweight in preterm births did not change in 1981–92 [2309 g (SD ± 664)] compared to 1971–80 [2308 g (SD ± 683)] in contrast to the first decade [2170 g (SD ± 732), *p *< 0.001].

The proportion of preterm births declined from 7% in 1961–70 to 6% in 1971–80 (Figure 5B and Table 2), but it increased again to 7% in 1981–92. In the last decade mean birthweight in all births increased despite the parallel increase in the percentage of preterm births. There was a two-fold increase in the percentage of caesarean section among preterm births from the early 1970s to the early 1990s, which partly accounted for this increase (Figure 5B).

Table 2 demonstrates that the gap between the most affluent and the most deprived groups of the population widened over the three decades.

## Discussion

Our study using population-based birth data in a single conurbation over three decades reported that between 1961 and 1992, when stillbirth and infant mortality rates declined dramatically, maternal age, parity, birthweight and gestational age changed substantially.

### Comparison with other studies

National trends on the total fertility rates for 1960–1990 mirror temporal trends shown in our study, where we used parity as a measure of fertility; during the 1960s 'baby boom', the national total fertility rates peaked in 1964 followed by a subsequent decline with a lowest level in the mid 1970s and a slight increase afterwards [24]. It has been suggested that the reduction in total fertility is attributable to improved means of fertility control (1967 Abortion Act and improved contraception efficacy) between 1967–68 and 1975. We also believe that the National Health Service (Family Planning) Act (1967), availability of free family planning services irrespective of age or marital status from April 1974, the Equal Pay Act 1970, the Sex Discrimination Act 1975 and the Employment Protection Act 1975, all contributed to women's reproductive decisions. This resulted in a decline in the proportion of teenage mothers and a parallel increase in the proportion of older mothers after the mid 1970s, as well as the overall increase in the mean maternal age in all and primiparous women. The increase in maternal age from the early 1980s was reported locally [6], nationally [25,26], in Europe [27] and in the United States [28,29]. Our data show that the mean maternal age in all and primiparous women was U-shaped with a declining trend from 1961 to the mid 1970s followed by a steady increase, repeating the national trend [24]. As advanced maternal age is associated with a higher risk of preterm birth and low birthweight [2,8], its rise from the mid 1970s reported here may have contributed to the observed increase in the percentage of preterm birth in the last decade. Thus a study suggested that delayed childbearing may play an increasingly important role in low-birthweight trends in the United States [30].

We report a steady increase in the overall mean birthweight starting from the mid 1970s, which we observed for term births only and despite the increase in the proportion of preterm births in the second half of the study period. Hence, the observed rise in the total mean birthweight is likely to reflect the increase in birthweight for gestational age for term infants. This was also observed in Norway, where an increasing trend was reported for term births for 1967–1998 [31], but not preterm (22–32 weeks) which were heavier in the first decade compared to the last, in contrast to our findings. Similar trends were also observed in Canada from 1981 to 1997 where the increase in mean birthweight was restricted to term infants [32]. A study based on the Northern Region of England population, with Newcastle as part of this population, reported that the increasing trend in higher birthweights continued in the 1990s [6]. An increase in mean birthweight has been also observed in other parts of England [33], nationally [34] and in other Western countries [31,32,35].

The proportion of preterm births declined in the second decade compared to the first, but it was followed by a steeper increase in 1981–92. To our knowledge, there are no population-based studies from the UK for comparison. Studies from other countries also reported the increase in the percentage of preterm birth from the 1980s [31,36,37]. Several factors may have contributed to this increase. Thus there was a two-fold increase in the percentage of caesarean section among preterm births from the early 1970s to the 1990s, as with advances in neonatal technology, survival of extremely preterm infants dramatically increased, which justified interventions for fetal or maternal indications at earlier gestational ages. Similarly, in Norway the increase in the percentage of preterm births was attributable to a dramatic increase in the percentage of caesarean section among births delivered between 28 and 35 weeks in the late 1980s-1990s compared to the 1960s-1970s [31]. The increase in births to older mothers, which are associated with a higher risk of preterm birth and a higher percentage of caesarean section due to a higher rate of complications of pregnancy, may also have contributed to this increase. Another factor may be a wider use of assisted reproductive technology in the UK from the late 1980s [38], which is associated with a higher risk of preterm birth in singletons [39,40] and is more widely used among older women.

Townsend deprivations scores, which we calculated for each birth in the database to measure neighbourhood socioeconomic status, also changed over time: the scores seemed to improve for the most affluent quintile and deteriorate for the most deprived, thus making the gap between the affluent and deprived groups wider. This is in line with the widening socio-economic and health inequalities which are now well documented in the UK.

### Strengths and limitations of the PAMPER birth record database

The population-based PAMPER birth record database contains historical high-quality birth data in a defined compact geographical setting over a 32 year period during which there have been significant changes in obstetric and neonatal services. The completeness of the PAMPER database both for the number of births and information collected for each birth is a major strength. National and local trends in the number of births in the UK confirm the temporal fluctuations also observed in the PAMPER study: the highest number of births at the beginning of the 1960s (a so-called 'baby boom'), followed by a decline in the 1970s and a further increase in the number of births during the 1980s [41]. The completeness of the data for the key variables described here is expressed in the low percentages of missing data for these variables.

The availability of accurate population-based gestational age, a major determinant of birthweight, is one of the leading strengths of the PAMPER database, as gestational age was not available in national birth statistics during the study period. Further, birthweight for live births was not collected in the UK at national level until 1975 (as part of the Child Health Births Notifications System). Without gestational age, interpretation of trends in birthweight could be misleading, as it is not possible to disentangle whether changes in birthweight are attributable to changes in rates of preterm birth or to changes in actual fetal growth. However, in the UK and elsewhere in the world there is a lack of information on the incidence of premature birth using accurate data by gestation [15].

The accuracy of the data for the key variables was ensured by multiple checking, internal (within the database) and external (with national and regional death data, and other local sources of birth record data) validation of the data.

The PAMPER database also has several limitations. The lack of information on some important determinants of fetal weight at birth such as maternal height, maternal smoking and exposure to environmental tobacco smoke, which have changed over time thereby affecting changes in birthweight, is disappointing. For example, an increasing trend in maternal height was reported in Scotland for 1980–2000 [42]. In the UK, the prevalence of smoking in women increased sharply during and after the Second World War, reaching the level of about 42–44% in the 1960s – early 1970s [43,44] followed by a gradual decrease thereafter [44]. However, adjustment for year of birth should be able to control for the effect of temporal changes in any factors influencing birth outcomes.

The accuracy of gestational age estimates is important for epidemiologic studies of pregnancy outcomes. Different methods for gestational age assessment (based on the last normal menstrual period (LMP) or early ultrasound measurements) throughout the study period may introduce bias in gestational age estimation over time. Thus it has been suggested that higher rates of preterm birth may be reported if determination of gestational age is based on ultrasonographic dating alone [45,46]. In the 1960s and 1970s, when gestational age estimate was based on LMP and, if the dates were uncertain, on the paediatric examination of the baby, it may have more uncertainty. However, while creating our birth record database, we made the recording of gestational age as objective and accurate as possible by accepting gestational age calculated from the recorded estimated date of delivery (EDD) (i.e. LMP based) for the majority of births rather than by entering gestational age recorded in the neonatal notes or birth records. For example, the percentage of gestational age records based on the recorded EDD for 1961–70 was about 87% of records with known gestational age. In this study the ultrasound age estimate has been used since the early 1980s only for pregnancies with uncertain date of LMP or if there was a significant discrepancy between the two estimates, therefore it should not bias gestational age estimates over time. Moreover, gestational age seems to be accurate in our study as birthweight distribution at early gestational ages has a single mode in contrast to other studies reporting bimodal birthweight distributions at early gestations with implausible high birthweights for gestational age [16,17].

## Conclusion

This historical population-based study documents substantial temporal changes in key maternal and fetal factors affecting birth outcomes over a 32-year period during which much social change has taken place. The availability of accurate gestational age is extremely important for correct interpretation of trends in birthweight.

## Competing interests

The authors declare that they have no competing interests.

## Authors' contributions

SG carried out the statistical analysis and drafted the paper. All authors were co-investigators on the Wellcome Trust grant, contributed to the initiation of the project and study design, and commented on the drafts of the paper. All authors have read and approved the final version of the manuscript.

## Pre-publication history

The pre-publication history for this paper can be accessed here:


